# ﻿*Carlephyton sajoreciae* (Araceae, tribe Arophyteae), a new species from Daraina, Northern Madagascar

**DOI:** 10.3897/phytokeys.265.165851

**Published:** 2025-10-20

**Authors:** Ranto Tiana Ratsiferanarivo, Haingotiana Johary Andrianjatovo, Franck Rakotonasolo, Rokiman Letsara, Mijoro Rakotoarinivo, Tao Wan, Sheng-Wei Wang, Neng Wei, Qing-Feng Wang

**Affiliations:** 1 State Key Laboratory of Plant Diversity and Specialty Crops, Wuhan Botanical Garden, Chinese Academy of Sciences, Wuhan 430074, China University of Antananarivo Antananarivo Madagascar; 2 University of Chinese Academy of Sciences, Beijing 100049, China Wuhan Botanical Garden, Chinese Academy of Sciences Wuhan China; 3 Department of Plant Biology and Ecology, Faculty of Sciences, University of Antananarivo, Antananarivo 101, Madagascar University of Chinese Academy of Sciences Beijing China; 4 Parc Botanique et Zoologique de Tsimbazaza, Antananarivo 101, Madagascar Parc Botanique et Zoologique de Tsimbazaza Antananarivo Madagascar; 5 Sino-Africa Joint Research Center, Chinese Academy of Sciences, Wuhan 430074, China Sino-Africa Joint Research Center, Chinese Academy of Sciences Wuhan China

**Keywords:** Arophyteae, *
Carlephyton
*, Daraina region, new species, taxonomy

## Abstract

*Carlephyton sajoreciae* is a new species of Araceae from northern Madagascar. It is distinguishable from the similar *C. darainense* mainly by the purple interior spathe, yellow spadix, 1-androus male flowers, loosely arranged stamens, and short, dark purple styles. A key for identification of the members of the *Carlephyton* has been provided. Interestingly, *C. sajoreciae* is the first species documented on humus-rich soils under forest, while other species grow on thin substrates among rocky outcrops. This finding highlights the botanical diversity in Madagascar, reinforcing the importance of conservation efforts in the region.

## ﻿Introduction

The family Araceae Juss. has been recognized as an early-diverging lineage within the monocots (Cabrera et al. 2008). Its classification has been refined through the integration of multiple data sources, including the monographic and morpho-anatomical dataset (Mayo et al. 1997), molecular sequence data (Cabrera et al. 2008), and restriction-site analyses (French et al. 1995), all of which have collectively contributed to the identification of distinct phenotypic characteristics within the family (Cusimano et al. 2011). Globally widespread and showing greater diversity in tropical areas, Araceae is represented in Madagascar by approximately 23 native and endemic species that fall into six genera, many exhibiting significant habitat specialization and morphological variation (Bogner 1975; Boyce et al. 2025). Among these, the tribe Arophyteae Bogner, which is endemic to Madagascar and characterized by a unique morphological set, i.e. globose pollen with spinose exine, female-male spadix zonation, and stamens connate or basally connate by their filaments (Bogner 1972); it comprises three small genera: *Arophyton*Jumelle (1928: 21), *Carlephyton*Jumelle (1919: 187), and the monotypic *Colletogyne*Buchet (1939: 23) (Mayo et al. 1998).

The genus *Carlephyton* is restricted to limestone outcrops in tropical deciduous forests, and it currently includes four species: *C. madagascariense*Jumelle (1919: 187), the first species described for the tribe amended by Buchet (1941), *C. diegoense*Bogner (1972: 18), *C. glaucophyllum*Bogner (1972: 15), with the most recently described species, *C. darainense*Bogner and Nusbaumer (2012: 210) in Daraina region of northern Madagascar.

A field investigation carried out in January 2025 in Daraina, within the Loky Manambato Protected Area in northern Madagascar, led to the discovery of a distinct *Carlephyton* population exhibiting unique morphological characteristics. Individuals were observed in mountain area within humid forest remnants and, at first sight, they are roughly similar to *C. darainense*. However, this population differs notably from it, raising the possibility of its recognition as a new species. Indeed, *Carlephyton* population in Daraina differs from other species in the genus by a combination of multiple morphological characteristics. Through integrated morphological studies, we further confirmed and described it here as a new species, named as *C. sajoreciae*.

## ﻿Material and methods

The morphological study was carried out by the observations and measurements on both living materials during fieldwork and dried specimens preserved in herbaria (DBEV, HIB, TAN; codes according to Thiers 2025), as well as the examination of digitized images from online sources, such as JSTOR Global Plants (https://plants.jstor.org), the Muséum National d’Histoire Naturelle (https://science.mnhn.fr/institution/mnhn/collection), and Kew Data Portal (http://www.data.kew.org). Morphological characteristics were observed and photographed with a Zoom Stereo Microscope-TS-10W stereomicroscope (PDV, China) and an EOS M5 Canon camera, and were measured using ImageJ (https://imagej.nih.gov/ij/). Morphology of pollen was observed and photographed with a HITACHI TM3030 scanning electron microscope (SEM) (Hitachi, Ltd., Japan).

## ﻿Results

The broad cordate-sagittate leaf shape of Daraina population resembles that of *Carlephyton darainense*. The inflorescence exhibits notable differences, characterized by a purple interior spathe surface and yellow spadix (Table 1). Although similar in morphology to *C. darainense*, the tuber is comparatively smaller than those found in *C. diegoense*, *C. madagascariense*, and *C. glaucophyllum*. Unlike all the species of the genus, Daraina population possess 1-androus male flowers, easily distinguishing it from previously described species. Furthermore, the arrangement of male flowers contrasts with that of *C. diegoense*, *C. madagascariense*, and *C. glaucophyllum*, which feature denser floral arrangements. The globose pollen grains are inaperturate with spinose exines as featured by all *Carlephyton* species (Grayum 1992).

**Table 1.** Morphological comparison of *Carlephyton sajoreciae* to its most morphologically similar species, *Carlephyton darainense*.

Character	*C. darainense*	*C. sajoreciae*
Petiole	14–22 cm long, flat above	9–13 cm long, ± flat above
Spathe	Externally and internally green, margins light green	Externally green with visible longitudinal veins, internally purple, dark purple margins
Spadix	2–3 cm long, light to deep purple	1.5–2.5 cm long, yellow
Male zone of spadix	1.5–2.0 cm long	1.0–1.5 cm long
Female zone of spadix	0.5–0.8 cm long, 5–14 flowers	0.5–0.7 cm long, 7–11 flowers
Male flower arrangement	Laxly arranged synandria along the male zone of the spadix	± loosely arranged free stamens along the male zone of the spadix
Stamens	2 stamens, connate at base, upper filaments free	1-androus
Filaments	0.8–0.9 mm long, upper free portion of filaments turned horizontally.	c. 0.5 mm long, ± upright
Style	Long, up to 2.5–3.0 mm tall, white	Short, c. 0.2 mm tall, dark purple

### ﻿ Carlephyton sajoreciae

Taxon classificationPlantaeAlismatalesAraceae

N.Wei, S.W.Wang, Q.F.Wang sp. nov.

A85FAA02-6E2D-52DF-80AC-912B60871EC7

urn:lsid:ipni.org:names:77370951-1

Figs 1, 2, 3

#### Type.

Madagascar • province of Antsiranana, sub-prefecture of Vohemar, rural commune of Daraina, (13°14'S, 49°37'E), in mountain area within humid forest remnants, alt. 324 m, 08 January 2025, *N. Wei & R.T. Ratsiferanarivo MADA-256* (holotype: HIB!; isotype: DBEV!).

#### Diagnosis.

*Carlephyton sajoreciae* is similar to *C. darainense* from which differs in bearing a single leaf (vs. 2–5 leaves arranged in a basal rosette in *C. darainense*), distinctive purple inner spathe surface (vs. green), yellow spadix (vs. whitish in the female zone and whitish to purple in the male zone), and 1-androus male flower (vs. synandria of two stamens).

#### Description.

Plant tuberous, with 1 leaf and 2–3 inflorescences. ***Tuber*** ± globular, 1.0–1.5 × 1–2 cm, brown, usually produces only one green leaf (Fig. 2F); ***roots***, small, white, arising on the upper side of tuber. ***Petiole*** 9–13 cm long, 3–5 mm in diameter, rounded below and ± flat above, ***cataphylls*** up to 6 cm long when basal. ***Leaf blade*** broadly cordate-sagittate, about 9–11 × 6.5–8.0 cm, green, apex acute to cuspidate with a 1–2 mm long mucro, base cordate to subhastate, basal lobes 3.0–4.5 cm long, sometimes slightly extrorse; ***venation*** reticulate, ***midrib*** well developed; ***primary lateral veins*** 4–5 on each side, ascending upwards and combining into a single vein 0.3–1.0 cm from margin, with a second collective vein located very close to margin; ***secondary lateral veins*** finer and located between the primaries; ***third-order veins*** much thinner and less visible, also present between the two collective veins. ***Peduncle*** 2.5–6.0 cm long, 1–3 mm in diameter, terete, white to light green. ***Spathe*** ovoid to slightly ellipsoid, 2.0–2.5 × 0.9–1.2 cm, externally green, with visible longitudinal veins (slightly darker), a noticeable dark purple margin along the slit where the spathe opens, and a purple inner surface, narrowed into a short, pointed apex or acuminate or mucronate tip (1 mm). ***Spadix*** 1.5–2.5 cm long, 3–4 mm in diameter in male zone (Fig. 3D), ***female zone*** 0.5–0.7 cm long, whitish, adnate to base of spathe, with 7–11 flowers, ***male zone*** 1.0–1.5 cm long, yellowish, ending in a short sterile, whitish to yellowish apex (2.0–2.5 mm); ***flowers*** unisexual, perigone absent; ***male flowers*** ± loosely arranged, synandria lacking, consisting of a single unit with one free stamen (1-androus), 0.6–0.8 mm tall; filaments short conoid, not very distinct, 0.1–0.3 mm in diameter, c.0.5 mm tall, thecae inserted at filament apex; ***pollen*** grains globular, 20 µm in diameter, inaperturate, with spiny crooked exines c. 1.5 µm long (Fig. 3G, H). ***female flowers*** 1.0–1.5 mm tall; ***ovary*** ± globular, cream to yellow in colour, unilocular, 0.8–1.0 mm in diameter, surrounded by small, yellow synandrodes (sterile male floral structures); ***style*** short, distinct, about 0.2 mm long, cylindric, dark purple, exceeding synandrodium, visibly elevating stigmas above ovary; ***stigma*** ± broad, discoid, 0.2–0.4 mm in diameter, white to light yellowish. ***Fruits*** ellipsoid when young, cream to yellowish, with a persistent brown stigma remnant.

#### Etymology.

The epithet “*sajoreciae*” is derived from the abbreviation of the authors’ affiliation, the Sino-Africa Joint Research Center (SAJOREC), Chinese Academy of Sciences, which was established in 2013 in Africa and Madagascar. The name is dedicated to the great contribution made by SAJOREC to biodiversity conservation in Africa and Madagascar over the past decade.

#### Distribution and ecology.

**Figure 1.**
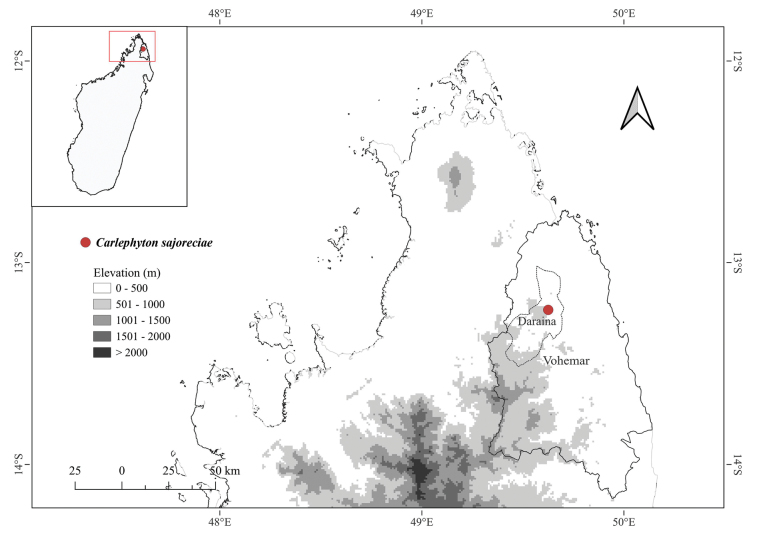
Known geographical distribution of *Carlephyton sajoreciae* in Loky-Manambato area, Vohemar district, SAVA region, Atsiranana province (Diego-Suarez), in northern Madagascar. The red dot indicates its only known locality.

This species has been observed on humus-rich surface soil layers, in humid, shaded forest understory habitats, occurring at mid-elevations on well-drained soils. It is only known from the type locality, the forest of Loky-Manambato area, Vohemar district, SAVA region, in the northern Madagascar (Fig. 1).

**Figure 2.**
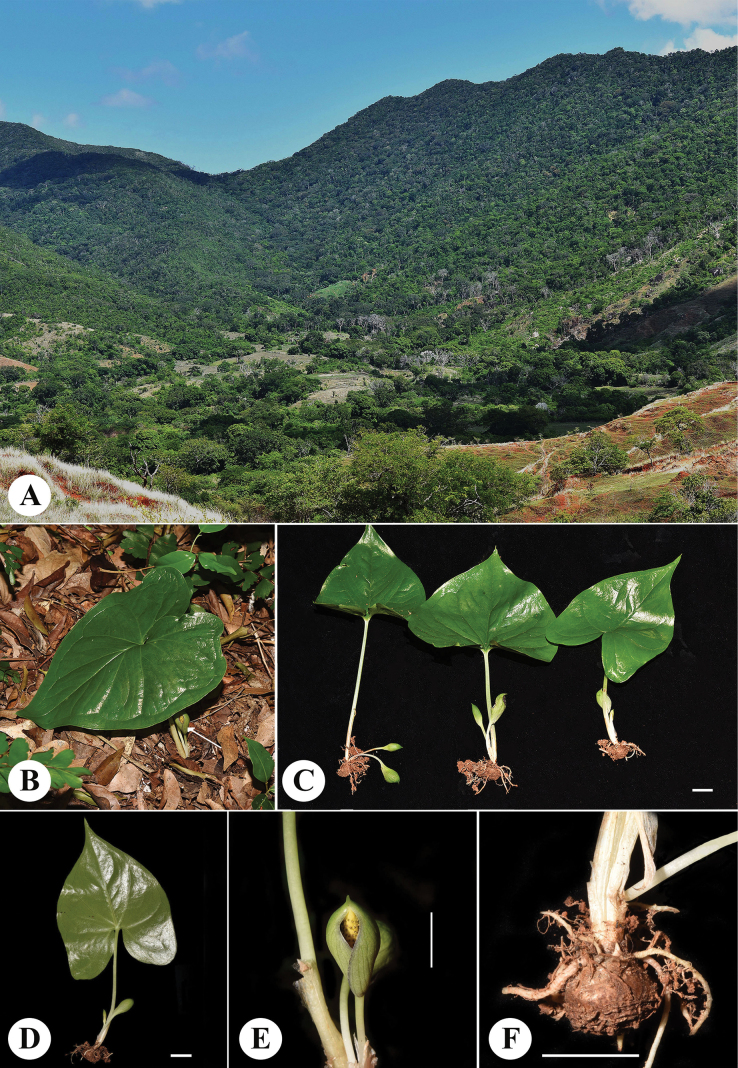
*Carlephyton sajoreciae*. A. Habitat; B. Plant in its natural habitat, arrow: inflorescence viewed from front; C. Whole individuals; D. Underside of leaf; E. Inflorescence that is not fully opened viewed from the front; F. Globular tuber with white roots. Scale bars: 1 cm (C–F).

**Figure 3.**
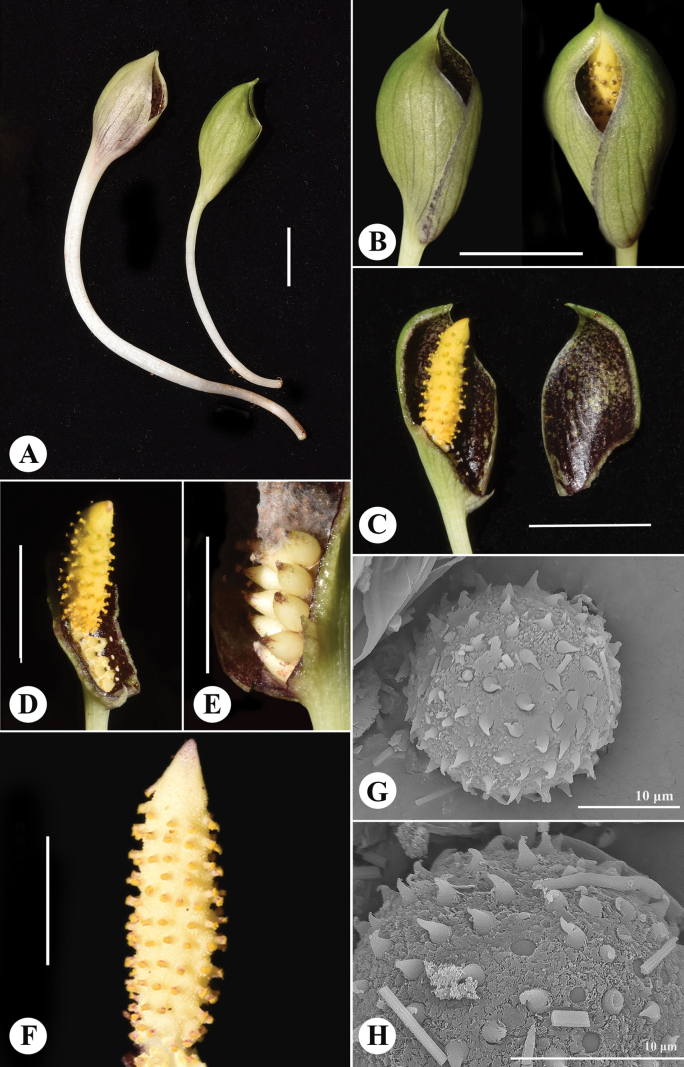
A, B. Inflorescence (not fully opened); C. Dissected spathe to show the purple interior; D. Close-up of the male and female spadix zone; E. Young fruits; F. Stamens of the male flowers, spadix ending in a short sterile apex; G, H. Crooked spiny exine globose pollen as seen by scanning electron microscope (SEM). Scale bars: 1 cm (A–D); 0.5 cm (E, F).

#### Phenology.

Flowering time in January.

#### Conservation status.

We found only one population, including ca. 40 individuals. Lacking further data, we prefer to assess the species as Data Deficient (DD) according to the IUCN Red List Categories and Criteria (IUCN).

#### Discussion.

The discovery of this new species highlights the rich biodiversity of Loky Manambato region and the need for further exploration, which have been indicated by the extensive number of new plant species recently discovered there (see e.g., Rakotonasolo and Davis 2024; Calvo and Callmander 2025; Darbyshire et al. 2025; Jongkind and Nusbaumer 2025).

*Carlephyton sajoreciae* is observed on humus-rich soil under forest, while other species in the genus grow on thin substrates among rocky outcrops (Bogner and Nusbaumer 2012). This new species shows a unique combination of morphological characters, making it easily distinguishable from the other species, i.e., the inflorescence with a purple interior spathe surface, the yellow spadix, the short, cylindric and dark style, which clearly elevates the stigma above the ovary. Unexpectedly, *C. sajoreciae* is distinctive in displaying 1-androus synandria devoid of any discernible evidence of synandrodium formation, while all other species have multistaminate synandria. This may reflect the loss of fused stamen characteristics, which have evolved into 1-androus flowers, as noted by Bogner (1972) for the monotypic genus *Colletogyne*.

Our finding emphasizes the presence of an unexplored habitat within the genus *Carlephyton*, pointing to a probable evolutionary adaptation to the shift of habitat. This establishes a starting point for future biogeographic studies of *Carlephyton* which will elucidate habitat shift patterns, thereby enhancing our understanding of the history of speciation and dispersal dynamics.

### ﻿Identification key to the species in genus *Carlephyton*, modified from Bogner and Nusbaumer (2012)

**Table:** 

1	Synandria completely connate and densely arranged; style short and narrowing towards the stigma; leaf blade pale green. Widespread in Northern Madagascar	***C. madagascariense***
–	Synandria connate only basally with upper part free or stamens free, densely, ± laxly or loosely arranged; style short or long; leaf blades green or glaucous	**2**
2	Female and male flower zones with bisexual flowers between them; synandria consisting of 2 stamens or reduced to 1 on upper part of the spadix; style conic; leaf blade glaucous. Massif de l’Ankarana	***C. glaucophyllum***
–	Female and male flower zones contiguous (no bisexual flowers occur between them); synandria consisting always of 2 stamens or 1-androus male flowers; style cylindrical, leaf blade green	**3**
3	Synandria with free part ± upright; margin of the synandrodium around the ovary lobed. Montagne des Français	***C. diegoense***
–	Synandria with free part turned horizontally or stamens free; margin of the synandrodium around the ovary entire	**4**
4	Synandria consisting always of 2 stamens, free part turned horizontally; style long, ± curved and white; spathe externally and internally green along the longitudinal central part and light green on the margins; spadix whitish and purple in male zone, whitish in female zone. Daraina region	***C. darainense***
–	No synandria present, male flowers 1-androus, stamens free; style very short (but distinct), not curved, dark purple; spathe externally green with purple margin and internally purple; spadix yellow in male zone and whitish in female zone. Daraina region	***C. sajoreciae***

## Supplementary Material

XML Treatment for Carlephyton sajoreciae

## ﻿Additional information

### Use of AI

No use of AI was reported.

### Author ORCIDs

Ranto Tiana Ratsiferanarivo https://orcid.org/0009-0003-1006-100X

Mijoro Rakotoarinivo https://orcid.org/0000-0001-6644-5107

Neng Wei https://orcid.org/0000-0002-0313-3912

### Data availability

All of the data that support the findings of this study are available in the main text.

## References

[B1] Bogner J (1972) Revision der Arophyteae (Araceae). Botanische Jahrbücher Fur Systematik 92: 1–63.

[B2] Bogner J (1975) Aracées. In: Humbert H (Ed.) Flore de Madagascar et des Comores.Muséum National d’Histoire Naturelle, Paris, 3–75.

[B3] Bogner J Nusbaumer L (2012) A new species of *Carlephyton* (Araceae) from northern Madagascar with notes on the species of this genus. Willdenowia 42(2): 209–217. 10.3372/wi.42.42206

[B4] Boyce PC Croat TB Hay A (2025) The Überlist of Araceae, totals for published and estimated number of species in aroid genera. 10.13140/RG.2.2.24068.64646

[B5] Buchet S (1939) Un nouveau genre malgache d’Aracées. Bulletin de la Société Botanique de France 86(1): 23–24. 10.1080/00378941.1939.10834145

[B6] Buchet S (1941) Sur deux Aracées endémiques de Madagascar. Bulletin de la Société Botanique de France 88(9): 846–849. 10.1080/00378941.1941.10834296

[B7] Cabrera LI Salazar GA Chase MW Mayo SJ Bogner J Dávila P (2008) Phylogenetic relationships of aroids and duckweeds (Araceae) inferred from coding and noncoding plastid DNA. American Journal of Botany 95(9): 1153–1165. 10.3732/ajb.0800073 21632433

[B8] Calvo J Callmander MW (2025) A New Species of the Malagasy Genus *Apodocephala* (Compositae). Novon: A Journal for Botanical Nomenclature 33(1): 24–28. 10.3417/2025969

[B9] Cusimano N Bogner J Mayo SJ Boyce PC Wong SY Hesse M Hetterscheid WLA Keating RC French JC (2011) Relationships within the Araceae: Comparison of morphological patterns with molecular phylogenies. American Journal of Botany 98(4): 654–668. 10.3732/ajb.1000158 21613165

[B10] Darbyshire I Callmander MW Randriamamonjy NJE (2025) A new species and a new combination in *Stenandriopsis* (Acanthaceae) from Madagascar. Kew Bulletin 80(1): 153–159. 10.1007/s12225-024-10246-9

[B11] French JC Chung MG Hur YK (1995) Chloroplast DNA phylogeny of the Ariflorae. Monocotyledons. Systematics and Evolution 1: 255–275.

[B12] Grayum MH (1992) Comparative external pollen ultrastructure of the Araceae and putatively related taxa. Missouri Botanical Garden 43: 1–167.

[B13] Jongkind CCH Nusbaumer L (2025) Two new species of *Combretum* (Combretaceae) from Loky Manambato (Daraina, north-east Madagascar). Candollea 80(1): 95–102. 10.15553/c2025v801a9

[B14] Jumelle H (1919) Les Aracées de Madagascar. Annales Du Musée Colonial de Marseille 7(1): 187.

[B15] Jumelle H (1928) Un nouveau genre malgache d’Aracées. Annales Du Musée Colonial de Marseille 6(2): 21–23.

[B16] Mayo SJ Bogner J Boyce PC (1997) The genera of Araceae. Kew Bulletin 53(2): 505. 10.2307/4114530

[B17] Mayo SJ Bogner J Boyce PC (1998) Araceae. In: Kubitzki K (Ed.) Flowering Plants Monocotyledons: Alismatanae and Commelinanae (except Gramineae).Springer, Heidelberg, Berlin, 26–74. 10.1007/978-3-662-03531-3_7

[B18] Rakotonasolo F Davis AP (2024) Four new deciduous species of *Hyperacanthus* (Rubiaceae: Gardenieae) from western Madagascar: the sofikomba alliance. Kew Bulletin 79(2): 345–358. 10.1007/s12225-023-10158-0

[B19] Thiers B (2025) [continuously updated]: Index Herbariorum: A global directory of public herbaria and associated staff. New York Botanical Garden’s Virtual Herbarium. http://sweetgum.nybg.org/ih/ [accessed: 06 October 2025]

